# Tattoo Regret, Complications, and Removal: A Cross-Sectional Study among Tattooed Individuals in Saudi Arabia

**DOI:** 10.1155/2024/5673785

**Published:** 2024-07-24

**Authors:** Hadeel Mitwalli, Nuha Alfurayh

**Affiliations:** ^1^ Department of Dermatology College of Medicine King Saud University, Riyadh, Saudi Arabia; ^2^ Department of Dermatology Imam Abdulrahman Alfaisal Hospital Ministry of Health, Riyadh, Saudi Arabia

## Abstract

**Background:**

A tattoo is a pigment permanently deposited into the skin to create different patterns. The worldwide percentage of tattooed individuals, as well as the level of tattoo regret, complications, and removal, has increased. However, data from Saudi Arabia are lacking.

**Objective:**

To determine the rates of tattoo regret, complications, and removal among tattooed individuals in Saudi Arabia.

**Methods:**

A cross-sectional study using an online survey was conducted in Riyadh, Saudi Arabia. A link to the survey was distributed through social media and contained twenty questions about participants' demographics, tattoo practices, regret, removal, and complications. Data were analyzed using the Statistical Package for Social Sciences (SPSS). A *P* value <0.05 was considered statistically significant.

**Results:**

A total of 181 tattooed individuals participated in the study. Most of them were females (95.6%), and 76.7% had their first tattoo at an age over eighteen years. A total of 47.5% had one tattoo session, and the most common body site was the eyebrows (57.5%). Fifty-eight percent regretted their tattoo, and 42.5% attempted to remove it. Upper extremity tattoos were significantly associated with regret (72.3%) (*P*=0.004) and removal (56.9%) (*P*=0.003). Tattoo removal was mostly due to cultural reasons (74%). Local itching (32%), pain (22.7%), and infection (10.5%) were reported and associated with tattoo regret (*P* < 0.05). Itching was significant in 65.4% of head and neck tattoos and 41.5% of upper extremity tattoos (*P* < 0.05). Participants were aware that laser is the best method for tattoo removal.

**Conclusion:**

Among tattooed individuals in Saudi Arabia, the rate of tattoo regret and removal is high, and the most reported complication is pruritus.

## 1. Introduction

Tattooing is the practice of depositing pigment into the dermal layer of the skin [1]. In the last several decades, the rate of tattooing has increased worldwide, particularly among young individuals [2–4]. There are different types of tattoo practices, including professional tattoos and cosmetic tattoos, such as eyebrows, eyeliner, and lip tattoos [5]. As tattoos have become more common, the rate of tattoo regret has subsequently risen [4]. An American study published in 2016 showed that 23% of tattooed individuals regret getting tattooed, in comparison to 14% in 2012 [6].

Several tattoo complications have been reported in the literature, including local and systemic infections, granulomatous reactions, scarring, photodermatitis, pseudolymphoma, and hypersensitivity reactions [7, 8]. Some patients with tattoo complications have reported a decreased quality of life [9].

As the practice of tattooing continues to grow in popularity, the desire for tattoo removal also grows [10, 11]. Multiple factors contribute to the rate of tattoo regret and the decision to have tattoos removed, including changes in lifestyle, occupation, or partner; tattoo color or shape; and tattoos received at an early age [12, 13]. Different modalities of tattoo removal have been studied with variable outcomes [5]. The most common and effective technique for tattoo removal is the quality-switched (QS) laser. It has demonstrated diverse outcomes depending on the parameters and techniques that have been used as well as the type of tattoo [10]. The most common lasers used for tattoo removal are neodymium-doped yttrium aluminum garnet (Nd-YAG) lasers at wavelengths of 532 nm and 1,064 nm, the QS 755 nm alexandrite laser, and the QS 694 nm Ruby laser [14]. Recently, picosecond lasers have become more favorable for tattoo removal [15].

To our knowledge, no study has investigated the rate of tattoo regret, complications, and removal among the Saudi population. This study aimed to determine the rates of tattoo regret, complications, and removal among tattooed individuals in Saudi Arabia.

## 2. Methodology

A cross-sectional study using an online survey was conducted. The survey items were based on those of previous similar studies, with modifications [4, 12]. The survey was translated into Arabic by a bilingual health science researcher and tested by two other bilingual health science researchers. A pilot study was conducted on fifteen randomly chosen participants to determine its comprehensibility, the time required to complete the survey, and any technical problems. The results of the pilot study are not included here. The study was conducted in Riyadh, Saudi Arabia. After obtaining the necessary approval from the Institutional Review Board of King Saud University, a link to the survey was distributed through different social media networks (Twitter, Snapchat, and Instagram) during the period from November 2022 to March 2023.

The final survey was composed of four sections. The first section included questions about basic demographics, age, gender, and nationality, and basic tattoo information, including the number of tattoo sessions, body sites of tattoos, and age at first tattoo. The second section contained questions to evaluate tattoo regret, including whether the participant regrets any of his or her current tattoos, their desire for tattoo removal at no charge, and their willingness to get a new tattoo. The third section included questions about tattoo complications, including itchiness, pain, infections, and other complications. Participants were requested to report tattoos that were pruritic or painful for more than 1 month after tattoo placement, as tattoos usually take 2-3 weeks to heal completely [12, 16]. The last section involved questions about tattoo removal, including attempts at removing any tattoo, removal sites, removal causes, to whom they would go for a tattoo removal, the most effective method of removing tattoos, and if they think the current methods can completely remove tattoos.

Data were analyzed using the Statistical Package for Social Sciences (SPSS) version 24. Frequency distributions were used for the numbers and percentages of qualitative variables. The chi-square test was used to compare the regret rate with other characteristics. Fisher's exact test was used instead of a two-by-two table that has a small sample size and where more than 20% of cells have expected counts less than 5. A *P* value <0.05 was considered statistically significant.

## 3. Results

A total of 181 tattooed individuals were included in the study, most of whom were females (95.6%) and Saudis (96.7%). More than two-thirds of the participants were aged between 18 and 35 years, and almost half of them (48.6%) got their first tattoo between the ages of 18 and 25 years. A total of 47.5% of tattooed individuals had only one tattoo session, 29.3% had two sessions, and 23.2% had three or more sessions. The most common sites of tattoos among the participants were the eyebrows (57.5%); upper extremities (34.9%), which included the hands, forearms, and arms, and the lower extremities (16.1%), which included the feet, legs, and thighs (Table 1).

More than half of the participants regretted their current tattoos (58%), would like to have one or more tattoos removed (66.3%), and were not considering getting a new tattoo within the next few years (62.4%). Of the participants who regretted their tattoos, 48.6% were aged between 18 and 25 years, 33.3% were aged between 26 and 35 years, 15.2% were aged between 36 and 45 years, and 2.9% were 46 years or older (*P*=0.241). Furthermore, 53.3% of participants who regretted their tattoos had their first tattoo between the ages of 18 and 25 years, 22.9% were between the ages of 26 and 35 years, and 13.3% were younger than 18 years (*P*=0.132).

**Figure 1:**
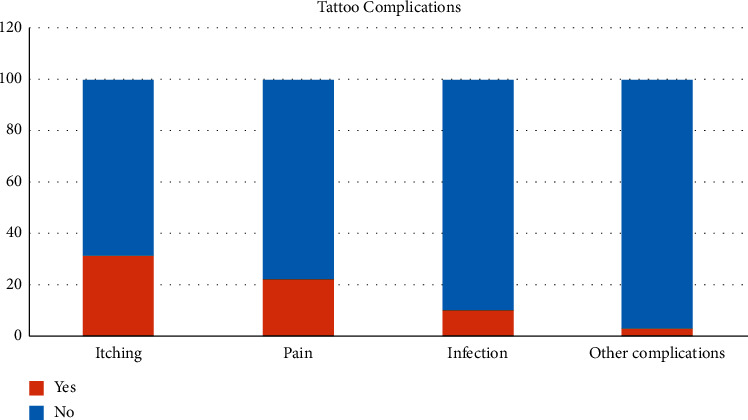
Rates of tattoo complications among 181 tattooed individuals in Saudi Arabia.

Various tattoo complications were reported by the participants, including skin itching (32%), local pain during or after receiving a tattoo (22.7%), cutaneous infection (10.5%), and other complications (3.6%), such as skin erythema, small pimples, peeling, and changes in tattoo color (Figure 1). No systemic complications were reported. Most tattoo complications were significantly associated with tattoo regret. A total of 43.8% of participants who experienced itching and 31.4% who experienced pain during or after the process regretted their tattoos. In addition, 15.2% of individuals who experienced an infection regretted their tattoos (*P*=0.014) (Table 2).

A total of 42.5% of participants attempted to remove one of their tattoos. The most common sites for tattoo removal were the eyebrows (49.4%), upper extremities (35%), and lower extremities (16.9%). The most common reasons for tattoo removal were cultural causes (74%), poor tattoo art (35%), and tattoos received at a young age (33.8%). Ninety-two percent of tattooed individuals chose a dermatology clinic as the appropriate place for tattoo removal, while 6% chose to visit a tattoo artist. A total of 93.9% of subjects believed that laser treatment was the most effective method for tattoo removal. In addition, 44.8% of respondents believed that the current medical methods available could completely remove tattoos with no traces or remnant pigmentation, while 37.6% did not. On the other hand, 17.7% of participants believed that current medical methods could remove tattoos completely before receiving their tattoos; however, their beliefs changed after receiving their tattoos (Table 3).

The association between tattoo location and regret, removal, and complications was investigated. For tattoos on the upper extremities, 72.3% of participants reported regret and 56.9% of participants reported removal (*P* < 0.05) (Table 4). Head and neck tattoos and upper extremity tattoos were significantly associated with itching in 65.4% and 41.5% of participants, respectively (*P* < 0.05). Individuals with tattoos on the eyebrows, neck and shoulders, and lower extremities also reported regret and tattoo removal (*P* > 0.05). Itching was also reported in tattoos on the lower extremities and head and neck (*P* > 0.05); other complications, including pain and skin infection, were also reported in tattoos placed at different body sites (*P* > 0.05).

## 4. Discussion

The present study has highlighted many important points regarding current tattoo practices and their consequences in Saudi Arabia. Traditionally, tattooing in Western countries was more prevalent among males; however, tattoo rates among females have increased in the last two decades, reaching a similar percentage in both genders [2, 3, 17]. Cosmetic tattooing has contributed to the increase in female tattoo rates [18, 19]. In our study, most participants were females, and the eyebrows were the most favored site for tattooing. This is consistent with the observed increase in eyebrow tattoos during and after the era of COVID-19 along with the mandatory wearing of face masks [20]. This can be explained by the fact that eyebrow makeup is time-consuming, and females are choosing to wear more permanent makeup using tattoos. Furthermore, we observed that most tattooed individuals received their first tattoo after the age of 18 years, which is consistent with previous studies that reported that small percentages of tattooed individuals received their first tattoo while they were of school age [12].

The literature demonstrates variable results concerning tattoo regret worldwide. An American study showed that 23% of adults with tattoos regret having a tattoo [12], and a study conducted in Turkey showed that 21.7% of participants wanted to remove their tattoos due to regret [21]. In other previous studies, it has been reported that 16–44% of people with tattoos regret at least one of them [22, 23]. A higher rate of tattoo regret was observed in our study. We investigated the factors that contributed to regret in tattooed individuals in Saudi Arabia. Patients who got their tattoos at an early age demonstrated more regret than those who were tattooed later in life, which is consistent with multiple previous studies [12, 21]. The eyebrows and upper extremities were the most commonly reported sites of tattoo removal in our study, with the latter being significantly related to regret and removal rates. Similarly, publications have shown that the most commonly reported sites of tattoo regret are the face and upper extremities [21]. This can be explained by the fact that the face and upper extremities are exposed more frequently than the lower extremities and trunk.

A wide spectrum of tattoo complications has been reported in the literature, ranging from mild erythema and pruritus to cutaneous malignancies [7, 13, 24, 25]. Pruritus, pain, and cutaneous infections were reported and significantly associated with tattoo regret in our study. Our finding supports the notion that tattoo complications play a role in tattoo regret [13]. Moreover, we reported a higher percentage of tattoo removal attempts than a study published in 2015 among 501 Americans, which reported that only 7% of participants attempted to remove one of their tattoos [12]. In the authors' opinion, the high rate of tattoo removal can be explained, to some extent, by the free access of Saudi citizens to health care facilities and the availability of tattoo removal lasers in most of the secondary and tertiary hospitals in Saudi Arabia. Cultural unacceptance and poor tattoo art were the top causes of tattoo removal in our study. However, in previous studies, not liking the tattoo anymore and the need to be eligible for an armed forces job were the most commonly reported reasons for tattoo removal [21, 26]. This difference is possibly due to the cultural variety among different study locations, and it suggests that the cultural unacceptance of tattoos plays a role in tattoo regret and removal.

To date, no treatment method has been identified to obliterate tattoos with no residues or adverse effects [14]. However, QS lasers are considered to be the most effective method of tattoo removal [14]. Participants who chose to remove their tattoos were aware that the most effective method for tattoo removal is laser therapy and that it should be performed by a dermatologist rather than a nonmedical professional such as a tattoo artist. To our knowledge, no study has tested the awareness of patients with tattoos regarding available removal methods. We found that only one-third of participants knew that the currently available methods might be unable to remove a tattoo completely with no residue or pigmentation. This lack of knowledge before getting a tattoo might have contributed to regret when removal attempts did not meet expectations.

In conclusion, this is the first study to report the rate of tattoo regret, complications, and removal among the Saudi population. Most tattooed individuals in Saudi Arabia have regretted their tattoos. The factors related to tattoo regret include placing a tattoo at an age younger than 25 years, developing cutaneous complications at the tattoo site such as pruritus and pain, and placing the tattoo at an exposed body site such as the face and upper extremities. We further report a high percentage of tattoo removal attempts where half of the individuals with eyebrow tattoos have attempted to remove them. Our findings demonstrated higher rates of tattoo regret and removal than previously reported worldwide.

A few limitations of the present study should be considered. First, although using an online survey helps to reach a larger number of the targeted sample than distributing paper surveys, people who do not have access to the Internet or do not prefer online surveys can be missed. Also, being self-administered takes a shorter time to collect the data than in-person interviews, yet reporting bias cannot be avoided. Second, the cross-sectional design of the study limits the clinical confirmation of tattoo complications, particularly infections, as well as identifying the causes of tattoo regret and removal. Lastly, the number of male participants and females older than 36 years included in the study was low. These gender and age biases are possibly due to the small sample size. The predominance of female participants while lacking the previous data on the male-to-female ratio of tattooed individuals in Saudi Arabia might affect the generalizability of the results.

We recommend longitudinal studies with larger sample sizes to include more males and females in all age categories using both online surveys and in-person interviews to overcome these limitations. Analysis of gender variations and cross-cultural comparisons are also recommended in future studies.

## Figures and Tables

**Table 1:** Demographics and basic tattoo information of 181 tattooed individuals in Saudi Arabia.

Demographic categories		*n*	%
Gender	Female	173	95.6
	Male	8	4.4

Age	18–25	81	44.8
	26–35	70	38.7
	36–45	24	13.3
	46 and above	6	3.3

Nationality	Saudi	175	96.7
	Non-Saudi	6	3.3

Age at first tattoo	18 or below	24	13.3
	18–25	88	48.6
	26–35	55	30.4
	36–45	11	6.1
	46 and above	3	1.7

Number of tattoo sessions	One	86	47.5
	Two	53	29.3
	Three or more	42	23.2

Body site of Tattoos^*∗*^	Eyebrows	104	57.5
	Upper extremities	65	34.9
	Lower extremities	30	16.1
	Neck and shoulders	26	14
	Others	17	9.4

^*∗*^One or more sites were selected per participant.

**Table 2:** The relation between the rate of tattoo regret and tattoo complications among 181 tattooed individuals in Saudi Arabia.

Tattoo complications			Regret		*P* value
			No (%)	Yes (%)	
Itching	No	*n*	64	59	<0.001^*∗*^
		%	84.2	56.2	
	Yes	*n*	12	46	
		%	15.8	43.8	

Pain	No	*n*	68	72	<0.001^*∗*^
		%	89.5	68.6	
	Yes	*n*	8	33	
		%	10.5	31.4	

Infection	No	*n*	73	89	0.014^*∗*^
		%	96.1	84.8	
	Yes	*n*	3	16	
		%	3.9	15.2	

^*∗*^Significant difference.

**Table 3:** Tattoo removal rate, causes, and practices among 181 tattooed individuals in Saudi Arabia.

	*n*	%
*Have you ever attempted to remove a tattoo?*		
No	104	57.5
Yes	77	42.5

*Site of removal* ^*∗*^		
Eyebrows	38	49.4
Upper extremities	27	35
Lower extremities	13	16.9
Lips	6	7.8
Face	4	5.2

*Causes of removal* ^*∗*^		
Cultural causes	57	74
Poorly done tattoo	27	35
Done at young age	26	33.8
Changes in tattoo color or shape with time	23	28.9
Personal maturity	22	28.6
Change of lifestyle or partner	8	10.4
Family pressure	5	6.5
Other causes	1	1.3

*If you are going to remove any of your current tattoos, to whom you would go?*		
Dermatologist	166	91.7
Tattoo artist	11	6.1
Pharmacist	3	1.7
Primary care physician	1	0.6

*Which of the following methods in your opinion is most effective in removing tattoo?*		
Laser	170	93.9
Topical medicine	8	4.4
Others (new technologies and special substance)	2	1.2

*Do you think the current methods can completely remove tattoos with no residual color or pigmentations?*		
Yes	81	44.8
No	68	37.6
Before I got a tattoo, I thought that current medical methods could remove tattoos completely, but it turned out not to be true	32	17.7

^*∗*^One or more choices were selected per participant.

**Table 4:** Rate of tattoo regret and tattoo removal according to tattoo site among 181 tattooed individuals in Saudi Arabia.

Tattoo site	Tattoo regret		*P* value	Tattoo removal		*P* value
	No	Yes		No	Yes	
Eyebrow	46	58	0.48	64	40	0.197
	44.2%	55.8%		61.5%	38.5%	

Neck and shoulders	10	16	0.69	14	12	0.69
	38.5%	61.5%		53.8%	46.2%	

Lower extremities	16	14	0.168	20	10	0.264
	53.3%	46.7%		66.7%	33.3%	

Upper extremities	18	47	0.004^*∗*^	28	37	0.003^*∗*^
	27.7%	72.3%		43.1%	56.9%	

^*∗*^Significant difference.

## Data Availability

The data that support the findings of this study are available from the corresponding author upon reasonable request.

## References

[1] Grumet G. W. (1983). Psychodynamic implications of tattoos. *American Journal of Orthopsychiatry*.

[2] Kluger N. (2015). Epidemiology of tattoos in industrialized countries. *Current Problems in Dermatology*.

[3] Laumann A. E., Derick A. J. (2006). Tattoos and body piercings in the United States: a national data set. *Journal of the American Academy of Dermatology*.

[4] Kluger N., Misery L., Seité S., Taieb C. (2019). Regrets after tattooing and tattoo removal in the general population of France. *Journal of the European Academy of Dermatology and Venereology: JEADV*.

[5] McIlwee B. E., Alster T. S. (2018). Treatment of cosmetic tattoos: a review and case analysis. *Dermatologic Surgery*.

[6] Shannon-Missal L. (2016). Tattoo takeover: three in ten Americans have tattoos, and most don’t stop at just one. *Harris Poll*.

[7] Kluger N. (2017). Cutaneous complications related to tattoos: 31 cases from Finland. *Dermatology*.

[8] Klügl I., Hiller K. A., Landthaler M., Bäumler W. (2010). Incidence of health problems associated with tattooed skin: a nation-wide survey in German-speaking countries. *Dermatology*.

[9] Hutton Carlsen K., Serup J. (2015). Patients with tattoo reactions have reduced quality of life and suffer from itch: dermatology Life Quality Index and Itch Severity Score measurements. *Skin Research and Technology*.

[10] Gurnani P., Williams N., Ghadah A. H. (2020). Comparing the efficacy and safety of laser treatments in tattoo removal: a systematic review. *Journal of the American Academy of Dermatology*.

[11] Naga L. I., Alster T. S. (2017). Laser tattoo removal: an update. *American Journal of Clinical Dermatology*.

[12] Liszewski W., Kream E., Helland S., Cavigli A., Lavin B. C., Murina A. (2015). The demographics and rates of tattoo complications, regret, and unsafe tattooing practices: a cross-sectional study. *Dermatologic Surgery*.

[13] Khunger N., Molpariya A., Khunger A. (2015). Complications of tattoos and tattoo removal: stop and think before you ink. *Journal of Cutaneous and Aesthetic Surgery*.

[14] Hernandez L., Mohsin N., Frech F. S., Dreyfuss I., Vander Does A., Nouri K. (2022). Laser tattoo removal: laser principles and an updated guide for clinicians. *Lasers in Medical Science*.

[15] Wu D. C., Goldman M. P., Wat H., Chan H. H. (2021). A systematic review of picosecond laser in dermatology: evidence and recommendations. *Lasers in Surgery and Medicine*.

[16] Fauger A., Sonck S., Kluger N., Chavagnac‐Bonneville M., Sayag M. (2022). Tattoo aftercare management with a dermo‐cosmetic product: improvement in discomfort sensation and skin repair quality. *Journal of Cosmetic Dermatology*.

[17] Muñoz-Ortiz J., Gómez-López M. T., Echeverry-Hernández P., Ramos-Santodomingo M. F., de-la-Torre A. (2021). Dermatological and ophthalmological inflammatory, infectious, and tumoral tattoo-related reactions: a systematic review. *The Permanente Journal*.

[18] Armstrong M. L., Saunders J. C., Roberts A. E. (2009). Older women and cosmetic tattooing experiences. *Journal of Women and Aging*.

[19] Straeteman M., Katz L. M., Belson M. (2007). Adverse reactions after permanent makeup procedures. *New England Journal of Medicine*.

[20] Tomita S., Mori K., Yamazaki H., Mori K. (2021). Complications of permanent makeup procedures for the eyebrow and eyeline. *Medicine*.

[21] Altunay İ. K., Güngör İ. E., Ozkur E. (2022). Tattoos: demographics, motivations, and regret in dermatology patients. *Indian Journal of Dermatology*.

[22] Lande R. G., Bahroo B. A., Soumoff A. (2013). United States military service members and their tattoos: a descriptive study. *Military Medicine*.

[23] Houghton S. J., Durkin K., Parry E., Turbett Y., Odgers P. (1996). Amateur tattooing practices and beliefs among high school adolescents. *Journal of Adolescent Health*.

[24] Shinohara M. M. (2016). Complications of decorative tattoo. *Clinics in Dermatology*.

[25] Wenzel S. M., Rittmann I., Landthaler M., Bäumler W. (2013). Adverse reactions after tattooing: review of the literature and comparison to results of a survey. *Dermatology*.

[26] Thakur B. K., Verma S. (2016). Tattoo practices in North-East India: a hospital-based cross-sectional study. *Journal of Cutaneous and Aesthetic Surgery*.

